# Not All Patients Need a CT When the Appendix Is Not Seen on Ultrasound: A Scoping Review

**DOI:** 10.3390/diagnostics16020304

**Published:** 2026-01-17

**Authors:** Ali Ramji, Justin J. Y. Kim, Gavin Low, Karim Samji, Mitchell P. Wilson

**Affiliations:** Department of Radiology and Diagnostic Imaging, University of Alberta, Edmonton, AB T6G 2R3, Canadalow1@ualberta.ca (G.L.);

**Keywords:** appendix, appendicitis, ultrasound, non-visualized, negative predictive value

## Abstract

**Background/Objective**: Recent North American guidelines suggest that CT is indicated for further evaluation where ultrasound (US) is negative, although the negative predictive value (NPV) of ultrasound in adult patients when the appendix is not seen remains unclear. To assess the negative predictive value (NPV) of ultrasound in adult patients when the appendix is not seen. **Methods**: A scoping review of MEDLINE and EMBASE was performed from inception to 13 May 2025 using PRISMA-ScR guidelines to identify studies evaluating the outcome of adult patients where the appendix is not seen on ultrasound, with preference for studies where there were no secondary signs of acute appendicitis (right lower quadrant free fluid, abscess, ileus, echogenic fat or regional lymphadenopathy). Original studies with at least 10 patients were included in the review. The reference standard included a combination of clinical follow-up, CT and/or pathology. Data synthesis was provided as a qualitative review of the existing literature. **Results**: Six studies were included in the review. The number of included patients range from 12 to 179 with a mean age of 29–38 years. Few studies reported the patient BMI. NPVs ranged from 80 to 90% for all indeterminate ultrasounds and 83 to 95% for studies where secondary signs of appendicitis were excluded (90 to 95% when non-surgical reference standards were included). Two studies reported NPVs of 96–100% when the pre-test probability was low. **Conclusions**: The NPV of indeterminate ultrasound for adult patients with right lower quadrant pain and no secondary signs of appendicitis is likely ≥90%. When combined with a low clinical suspicion, the NPV is likely >95%. The appropriateness of a subsequent CT indication when the appendix is not visualized on ultrasound should be determined on an individualized basis.

## 1. Introduction

The American College of Radiology (ACR) and Canadian Association of Radiologists (CAR) have recently published updated and new guidelines, respectively, on imaging adult patients with right lower quadrant pain [1,2,3]. The ACR guideline indicates that CT is “usually appropriate” while ultrasound (US) “may be appropriate” as an initial imaging tool for adult patients with right lower quadrant pain [1]. By contrast, a recently published CAR guideline recommends ultrasound as the initial study of choice in most young adults with a body mass index (BMI) < 25 kg/m^2^, with CT indicated for further evaluation where ultrasound is negative [3]. The cited range of non-visualized appendix with ultrasound was noted to be between 15 and 84% of cases and is sonographer and radiologist dependent, with a retrocecal positioned appendix being the most difficult to visualize [3,4,5,6]. Both sets of guidelines are focused on the emergency department setting and do not recognize other clinical environments where CT may be less readily available, nor do they stratify patients by pre-test probability of disease. In circumstances where CT may not be readily available and/or pre-test probability of disease is deemed to be low based on clinical and biochemical parameters, the use of ultrasound may be the preferred initial modality and a negative or indeterminate ultrasound may still serve as an acceptable clinical standard for excluding appendicitis and other significant causes of right lower quadrant pain in appropriate patients [7]. To address this controversy, we performed a scoping review evaluating the negative predictive value (NPV) of ultrasound in adult patients with right lower quadrant pain where the appendix was not visualized, preferentially identifying studies with patients demonstrating no secondary signs of acute appendicitis and/or a low pre-test probability was established.

## 2. Materials and Methods

This study was performed in accordance with the PRISMA extension for scoping reviews (PRISMA-ScR) [8]. An a priori protocol was developed but not registered and is available upon request from the corresponding author. The study sought to identify original articles evaluating the negative predictive value of ultrasound for excluding appendicitis and other significant pathologies in adult patients (≥18 years) with right lower quadrant pain when the appendix was not seen, preferentially when there were no secondary signs of appendicitis and/or a low pre-test probability was established. Secondary signs of acute appendicitis on ultrasound were defined as free fluid, abscess, ileus, echogenic fat and regional lymphadenopathy (≥1 cm in short axis) in the right lower quadrant. No thresholds were established to define low pre-test probability patients, with data included as a supplementary qualitative note if reported in an included study. The reference standard was a combination of clinical follow-up (minimum 1 month), CT follow-up imaging and/or pathology. The use of non-surgical reference standards, including CT and/or clinical follow-up, was necessary to limit the verification bias associated with surgical pathology, which is often reserved for patients with a high clinical suspicion. Original studies with a minimum of 10 cases meeting the criteria were included in the qualitative review. All non-original articles (such as review articles and guidelines) and case studies or series with <10 patients were excluded.

### 2.1. Literature Search

A comprehensive search of MEDLINE, EMBASE and the Cochrane Library for Reviews was performed by one author with extensive experience in systematic and scoping reviews and 10 years of imaging experience (MPW). The search was performed from inception until 13 May 2025. Combinations of title/abstract/keywords or medical subject heading terms including “ultrasound” AND “appendix” AND “adult” was individualized by database and are shown in Table 1. The search terms were intentionally broad to avoid excluding studies accidentally in each database. No language restrictions were applied. A gray literature search including the most recent year of the Radiological Society of North America (RSNA) annual meeting and the most recent two years of the American Roentgen Ray Society (ARRS) annual meetings were also performed. Search results from each database and the gray literature were combined and duplicates were removed. Title and abstracts were then screened for relevance by one experienced author with 10 years of imaging experience (MPW). A full text review was subsequently performed by another author with one year of experience in systematic reviews and data extraction (AR). Reference lists from included studies were screened to identify additional relevant articles, which were also reviewed in full text if deemed relevant.

### 2.2. Data Extraction

One author performed the initial data extraction from included articles (AR). A more experienced author re-reviewed key results for determining negative predictive values in each article (MPW) and any discrepancies were resolved by consensus. Extracted content included first author, publication year, country of origin, study design (prospective versus retrospective), presenting setting, number of patients with a non-visualized appendix, mean age, age range, sex distribution (male: female), mean BMI (kg/m^2^), reporting standards of secondary signs, number of patients without appendicitis, number of patients with appendicitis, NPV, reference standard, ultrasound vendor and transducer frequency, technician experience, and other individually relevant comments by study.

### 2.3. Data Analysis

A summary table and qualitative review of the original data was performed. A risk of bias assessment was not performed as this is not required for scoping reviews.

## 3. Results

**Figure 1:**
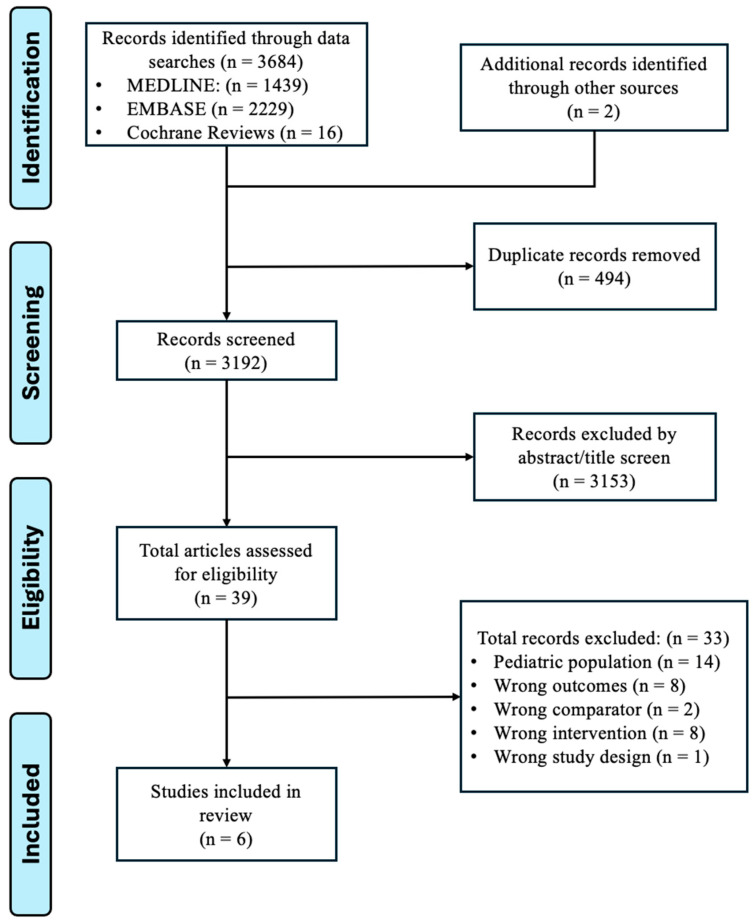
PRISMA flow diagram of included studies evaluating the negative predictive value of ultrasound in adult patients when the appendix was not seen.

A total of 3176 were identified from the database search and reference checks after duplicate removal. Of these, 39 were reviewed in full text, and 6 studies were included in the review [7,9,10,11,12,13]. One included study was an unpublished conference abstract [13]. The PRISMA flow diagram is shown in Figure 1.

Summary study characteristics are shown in Table 2. Study publication dates ranged from 2004 to 2024, were each performed in a different country, and were predominantly retrospective studies of emergency department patients. The number of included patients ranged from 12 to 179 with mean ages between 29 and 38 years. Two studies included one patient < 18 years of age but were deemed adequate for inclusion as it was felt this would not alter overall results [10,11]. One study excluded obese patients while most studies did not comment on BMI [7]. Most studies used a combination reference standard. All patients in one small study of 12 patients underwent surgery with pathological confirmation, even for cases where the appendix was not seen and there were no secondary signs of appendicitis on ultrasound [10]. Most technicians were either experienced sonographers or staff radiologists across all studies.

Negative predictive values across all studies ranged from 80 to 90%. Three studies explicitly excluded secondary signs of acute appendicitis and reported the following NPVs: Koname et al.: 83% (10/12); Jones et al.: 90% (107/119); Brahm et al. (unpublished abstract): 95% (specific numerator/denominator details are not documented) [7,10,13]. Two studies reported NPVs of the non-visualized appendix when combined with a clinical and biochemical scoring system (Alvarado score) with NPVs of 96% when the Alvarado score was <6 [9] and 100% when the Alvarado score was <4 [7].

## 4. Discussion

When using studies which include a non-surgical reference standard, this scoping review indicates that the NPV for excluding appendicitis and other significant pathologies of the right lower quadrant in adult patients undergoing ultrasound for this purpose where the appendix was not seen but there are no other secondary signs of appendicitis is likely ≥90%. Further, when combined with low clinical and biochemical concern for appendicitis (ex: Alvarado score < 6), the NPV is likely >95% in this setting; however, this estimate should be interpreted cautiously given the small number of studies and the inclusion of non-peer-reviewed evidence in the form of an unpublished abstract. This study supports an individualized recommendation for further evaluation and/or management, where some patients may be adequately surveyed clinically, even when the appendix is not seen.

A brief description of the six original analyzed studies is included below, each in a separate paragraph. This provides baseline clinical information for which the scoping review was based on.

Ulutas et al. [9] performed a retrospective study to assess the clinical, imaging and pathology results of adult patients with a preliminary diagnosis of acute appendicitis in the emergency department setting, in whom the appendix was not visualized on ultrasound. Pregnant and obese patients were excluded as these characteristics decrease the diagnostic value of ultrasound. In total, 176 patients were included, of which 79 (44.9%) underwent CT. Appendicitis was identified in 13 (16.5%) cases on CT, with the rest being normal or attributed to alternative causes such as ovarian cyst rupture or ureterolithiasis. Of the 97 (55.1%) patients who did not undergo CT, 2 patients returned with a diagnosis of appendicitis. A total of 21 (11.9%) patients underwent surgery with pathological evaluation, of which 6 (28.6%) did not have appendicitis. In combination, a total of 161 patients with a non-visualized appendix did not have a diagnosis of appendicitis, yielding an NPV of 90%. This study found that an Alvarado score of ≥6 and leukocytosis were predictive of appendicitis. An Alvarado score of ≥6 was observed in 80% of patients with appendicitis compared to 4.3% in those without the disease. The authors concluded that further imaging with CT or MRI can be avoided in patients with a non-visualized appendix on ultrasound if the Alvarado score is <6 and there is no leukocytosis.

Jones et al. [7] assessed the value of a low Alvarado score in predicting appendicitis in adult patients who underwent a non-diagnostic ultrasound in the emergency department setting. This study’s reference standard consisted of CT, supplemented by pathological examination when available. In total, 107 of the 119 patients who underwent CT did not have appendicitis, giving an NPV of 90%. Appendicitis was diagnosed in 12/70 (17.1%) patients with an Alvarado score of ≥4, compared to 0/49 patients with a score of ≤3. There were 14 (11.8%) patients who underwent surgery with pathological evaluation, of which 2 (14.3%) did not have appendicitis. The authors concluded that further imaging with CT can safely be avoided in adult patients with a non-diagnostic ultrasound if there is an Alvarado score of ≤3.

Kouamé et al. [10] performed a prospective study to assess the value of indirect signs in diagnosing appendicitis on ultrasound when the appendix was not seen. A total of 172 patients (28%) were reported to have a non-visualized appendix. Of these, only 12 (7%) had no secondary signs of appendicitis and were therefore included in this review. Pathological examination served as the reference standard in this study. Among the 12 patients with a non-visualized appendix and no secondary signs of appendicitis, 10 were confirmed to not have appendicitis, corresponding to an NPV of 83%. All 12 patients underwent surgery with pathological evaluation. The authors examined three indirect signs of appendicitis, including pain on probe compression, hypertrophy of peritoneal fat and localized hypokinesia of bowel loops. The presence of all three signs carried a sensitivity, specificity and positive predictive value (PPV) of 83.9%, 85.7% and 95.7%, respectively, when the appendix was not seen. The authors concluded that the presence of these indirect signs can be helpful in predicting appendicitis in patients with a non-visualized appendix when CT is unavailable.

Koseekriniramol et al. [11] performed a retrospective investigation to assess the utility of various clinical and imaging factors in predicting appendix visualization on ultrasound in adult patients with suspected appendicitis in the emergency department setting. The reference standards used in this study included CT, pathological examination or clinical follow-up. There were 59 (69%) patients with a non-visualized appendix, of which 47 did not have appendicitis, yielding an NPV of 80%. All 12 cases of appendicitis were confirmed on pathological evaluation following surgery. The remaining 47 negative cases were established through a combination of CT, pathological examination or clinical evaluation, although the exact proportion is not stated. The authors reported no association between appendix visualization on ultrasound and the patient characteristics studied, which included age, sex, weight, BMI, duration of symptoms, Alvarado score and abdominal wall thickness.

Kessler et al. [12] conducted a prospective study to assess the sensitivity, specificity, accuracy, PPV and NPV of ultrasound, Doppler ultrasound and laboratory findings in the diagnosis of acute appendicitis. The reference standard consisted of either pathological examination or clinical follow-up. There were 21 (17%) patients in whom the appendix was not visualized on ultrasound. Of these patients, 19 did not have appendicitis, corresponding to an NPV of 90%. The two cases of appendicitis were confirmed on pathological evaluation following surgery. The remaining 19 negative cases were established through a combination of pathological evaluation or clinical follow-up, although the exact proportion is not stated, which is a notable limitation of this study. Another limitation of this study is the lack of demographic information specifically related to the patients with a non-visualized appendix. In patients with a visualized appendix, the authors found that a diameter of ≥6 mm had a sensitivity, specificity, PPV and NPV of 98%, making it the most accurate appendiceal finding for appendicitis. Regarding laboratory findings, they reported a PPV of 71% when both the CRP and WBC were elevated, and an NPV of 66% when these values were normal.

In an unpublished conference abstract by Brahm et al. [13], the authors assessed the NPV of a non-visualized appendix on ultrasound in patients with a suspected diagnosis of appendicitis in the emergency department setting. Secondary signs of appendicitis were also documented. The reference standard consisted of CT with or without pathological examination. The exact number of patients who underwent surgery was not provided, which is a notable limitation of this abstract. In total, 116 patients were included in this study, of which 97 did not have a diagnosis of appendicitis, yielding an NPV of 84%. This value increased to 95% when secondary signs of appendicitis were excluded.

This scoping review was limited to the quality of the underlying data. The included studies were heterogeneous in several aspects, including study design, practice setting, patient selection criteria, definitions of a non-visualized appendix and of secondary signs. Also, many of the individual cohorts were relatively small, resulting in imprecise estimates with wide confidence intervals. As most of the studies were single-center retrospective series, often with non-consecutive sampling, this introduces potential selection bias and may overestimate diagnostic performance in centers with specific expertise or lower appendicitis prevalence. This heterogeneity precludes a formal meta-analysis; therefore, our summary estimates should be interpreted as approximate rather than precise pooled values. Taken together, the reported NPVs are an approximate observation rather than a definitive benchmark which supports the possibility that a CT can be safely deferred in adult patients with a non-visualized appendix with no secondary signs. However, this does not justify a guideline recommendation to reduce CT use in all adults with right lower quadrant pain. This data is best interpreted as reinforcing an individualized approach with decision regarding subsequent CT-guided by pre-test probability and existing clinical pathways. Most studies were retrospective single institution reviews with only two studies providing a complete data set of adult patients where the appendix was not seen and secondary signs of appendicitis were explicitly excluded [7,10]. One of these studies included only 12 patients and was likely a biased patient selection as all patients went to surgery regardless of imaging findings [10]. The other larger study of 119 patients showed an NPV of 90% [7]. An unpublished conference abstract indicated an NPV of 95% when secondary signs were excluded but did not provide specific data [13]. The inclusion of this unpublished conference abstract with incomplete numeric data represents a limitation of our review which results in the precision and generalizability of this NPV estimate as uncertain. The purpose of including this abstract is for supportive evidence, rather than being a driver of our conclusions. Despite the low confidence of this abstract, its results are supported by a larger excluded study evaluating both pediatric and adult patients where the appendix was not seen and there were no secondary features showing an NPV of 98% (225/229) [14]. Taken in combination, we believe it is reasonable to conclude that the NPV is likely ≥90% in this scenario. The absence of secondary signs of appendicitis when the appendix is not seen is also shown to have a high NPV on CT, with an NPV nearing 100% (45/46) [15]. This conclusion is further supported by the remainder of the studies which show an NPV ranging from 80 to 90%, even when secondary signs of appendicitis were not specifically excluded. This is also supported by other larger excluded studies evaluating both pediatric and adult populations where NPVs of 87% (411/473) and 94% (216/229) were obtained without excluding secondary signs of appendicitis [5,14].

A higher confidence for excluding appendicitis when the appendix is not seen can be made when the pre-text probability is low. Two included studies showed NPVs > 95% for this scenario, even if secondary signs were not overtly excluded [7,9]. Specifically, one study found an NPV of 96% in patients with an Alvarado score < 6 [9]. Another study found an NPV of 100% when the Alvarado score was <4 [7]. Similarly, another excluded study of combined pediatric and adult patients showed an NPV of 96% (50/52) for indeterminate ultrasound cases with a low degree of clinical and biochemical concern (Alvarado score < 5) [16]. Taken in combination, we believe it is reasonable to conclude that the NPV is likely ≥95% in this scenario.

Limited details on BMI were provided in the study of 119 patients noted above, specifically excluding obese patients [7]. This is a significant limitation of the existing evidence, as it prevents the evaluation of ultrasound’s diagnostic performance for appendicitis in patients with an elevated BMI. It is well recognized that increased body habitus can reduce ultrasound penetration and degrade image quality, making appendix visualization challenging. This lowers diagnostic performance in obese patients. Because only a minority of cohorts provided BMI data, and none stratified appendix visualization or NPV rates by BMI categories, our dataset does not allow for definitive conclusions based on patients with higher BMI. Thus, we agree with current recommendations to proceed to CT in appropriate patients with elevated BMI as the data does not appear to be adequately established in these patients [3]. To address this issue, future avenues of research are needed to assess the NPV of ultrasound for appendicitis in patients with a high BMI. While both American and Canadian guidelines indicate that CT may be more appropriate in patients ≥ 30 years of age, no clear evidence in this review has shown a less favorable evaluation of the appendix with ultrasound in patients ≥ 30, with one study suggesting that age does not contribute to appendix visualization [11]. Again, the appropriateness of a CT first recommendation for patients ≥ 30 should be determined on a multi-factor basis including availability of CT, patient BMI and other comorbidities and informed decision making in consort with the patient. Some patients ≥30 may still benefit from an ultrasound first approach [1]. The clinical relevance of a high NPV for a non-visualized appendix also depends on the healthcare setting, radiation exposure and costs. In situations where radiation exposure should be minimized, or access to CT scanners is limited, whether due to location or cost-limitations, an NPV ≥ 90% may be more influential in supporting a negative diagnosis of appendicitis.

This review was also not designed to specifically evaluate differences in NPV by sex or pregnancy status. One study showed no difference in the visualization of the appendix between genders [11]. No conclusions can be made about the appropriateness of ultrasound only imaging in indeterminate cases during pregnancy and we therefore agree with current guidelines to proceed with MRI (or low dose CT when MRI is unavailable) in these cases.

Future prospective studies are needed to determine the NPV of a non-visualized appendix on ultrasound with a clearly defined pre-test probability, which may include clinical scores, such as the Alvarado or Appendicitis Inflammatory Response scores, specific clinical presentation, and laboratory findings. This would allow for a more accurate NPV that is adjusted to patients with different pre-test probabilities. Additional related areas to explore in future studies include evaluating the NPV in pregnant patients and determining the quantifying the impact of BMI and AI-assisted ultrasound on appendix visualization and NPV.

## 5. Conclusions

This scoping review, which is limited by the quality of available literature, suggests that ultrasound can be used to exclude appendicitis and other significant pathologies of the right lower quadrant in appropriate adult patients, even when the appendix is not visualized. In patients where no secondary signs of appendicitis are present, the NPV is likely ≥90% and increases to >95% when there is also a low pre-test probability for disease. The appropriateness of a subsequent CT indication when the appendix is not visualized on ultrasound should be determined on an individualized basis.

## Figures and Tables

**Table 1:** Search strategy by database.

Database	Search Terms
MEDLINE	(ultrasound.mp. OR exp Ultrasonography) AND (exp Appendicitis OR appendicitis.mp. OR appendix.mp. OR exp Appendix/) AND (exp Young Adult/OR exp Adult OR adult.mp)
EMBASE	(exp ultrasound/OR ultrasound.mp) AND (exp appendicitis/OR exp acute appendicitis/OR appendicitis.mp. OR exp appendix/OR appendix.mp.) AND (adult.mp. OR exp adult/)
Cochrane Reviews	appendix AND ultrasound

**Table 2:** Summary details of included studies evaluating the negative predictive value of ultrasound for adult patients when the appendix was not seen.

Author	Publication Year	Country	Study Design	Presenting Setting	No. Patients	Mean Age (Range)	Sex Distribution (M/F)	Mean BMI (kg/m^2^)	Secondary Signs Excluded?	Patients Without Appendicitis	Patients with Appendicitis	NPV	Reference Standard	Ultrasound Vendor	Ultrasound Transducer Frequency	Technician Experience	Additional Comments
Ulutas et al. [9]	2025	Turkey	Retrospective	ED	176	38 (18–89)	113 (64.2%)/63 (35.8%)	NR	NR	161	15	90%	CT ± pathologic examination	NR	NR	NR	NPV = 96% when Alvarado score <6
Jones et al. [7]	2015	United States	Retrospective	ED	119	29 (19–69)	26 (28%)/93(72%)	Obese patients excluded	Excluded	107	12	90%	CT ± pathologic examination	Siemens or General Electric	NR	Sonographers (5+ years of experience); reviewed by radiology residents or staff	NPV = 100% when Alvarado score < 4
Kouame et al. [10]	2012	Cote d’Ivoire	Retrospective	NR	12	29 (15–45) *	NR	NR	Excluded	10	2	83%	Pathologic examination	General Electric	3–11 MHz	Staff Radiologists	All patients underwent surgery in this study
Koseekri-niramol et al. [11]	2015	Thailand	Retrospective	ED	59	37 (16–86) *	13 (22%)/46 (78%)	22	NR	47	12	80%	Clinical follow-up (3 months), CT and/or pathologic examination	Philips	3–5 MHz	Staff Radiologist or senior Radiology resident	Sex, Alvarado score, appendix location, age, weight, and duration of symptoms were not associated with visualization of appendix in this study
Kessler et al. [12]	2004	France	Retrospective	ED	21	NR	NR	NR	NR	19	2	90%	Clinical follow up or pathologic examination	Siemens	5–10 MHz	Staff Radiologists with 2, 5 or 10 years of experience	
Brahm et al. [13]	2011	NR	Retrospective	ED	116	NR	NR	NR	Included in total population **; Excluded population reported	97	19	84% ***	CT ± pathologic examination	NR	NR	Experienced abdominal radiologists	NPV = 95% when no secondary features were present (patient numbers not provided in abstract)

* Study deemed adequate for inclusion as only one patient < 18 years of age was included. ** Reported secondary signs included: echogenic fat, free fluid and local lymphadenopathy. *** Negative predictive value was 95% when secondary signs of appendicitis were excluded (patient numbers not provided in abstract). Legend: ED = Emergency Department; NR = not reported; No. = number; NPV = negative predictive value; CT = computed tomography.

## Data Availability

No new data were created or analyzed in this study. Data sharing is not applicable to this article.
